# Development of Molecular Marker Linked to Seed Hardness in Pomegranate Using Bulked Segregant Analysis

**DOI:** 10.3390/life13051123

**Published:** 2023-05-01

**Authors:** Keziban Yazıcı, Betül Gönülkırmaz, Mehtap Şahin Çevik

**Affiliations:** 1 Department of Horticultural Sciences, Faculty of Agriculture, Recep Tayyip Erdoğan University, Rize 53300, Turkey; 2 Department of Agricultural Biotechnology, Faculty of Agriculture, Ispara University of Applied Sciences, Isparta 32260, Turkey

**Keywords:** BSA, InDel markers, MAS, marker assisted selection, molecular breeding, RAPD

## Abstract

The pomegranate (*Punica granatum* L.) is one of the fruit species with the oldest cultural history. There are many traits to determine the quality of pomegranate fruits. Among them, soft-seeded feature of pomegranate fruit is important trait for the market value of the fruit. For this reason, the demand for pomegranate varieties with soft seeds has been increasing, especially in recent years. In this study, molecular markers associated with seed hardness were developed to distinguish pomegranate cultivars with soft-seeded feature based on genomic DNA at the early stages of the pomegranate breeding process. For this purpose, pomegranate genotypes and/or cultivars from the population involved in reciprocal crosses of hard-seeded Ernar, medium-hard-seeded Hicaznar, and soft-seeded Fellahyemez cultivars were grouped as soft-seeded or hard-seeded. Further, leaf samples were collected from individuals belonging to each group. Then, the genomic DNA was isolated from each plant separately, and equal amount of genomic DNA from individuals with the similar seed hardness were mixed for bulked segregant analysis (BSA). The bulked genomic DNAs of opposite characters were analyzed by polymerase chain reaction (PCR) using random decamer primers to develop random amplified polymorphic DNA (RAPD) markers associated with soft-seeded or hard-seeded pomegranates. A total of three RAPD markers were determined to distinguish the individuals having soft- or hard-seeded pomegranate genotypes and/or cultivars. As a result of the comparison of the DNA sequences of these RAPD markers, insertion-deletions (inDels) primers were designed to developed and validate a PCR assay to distinguish the soft- and hard-seeded pomegranate genotypes/cultivars from each other. The molecular markers developed in this study will enable us to distinguish soft-seeded pomegranate types easily in a short time at the early stages of the pomegranate breeding programs.

## 1. Introduction

Pomegranate, one of the ancient fruit species, has been consumed both fresh and juiced for thousands of years. The demand for the production and consumption of pomegranate fruit has been increased in recent years due to its important bioactive properties such as antimicrobial [1], antiparasitic [2], antiviral [3], and anticancer [4] activities. One of the most important reasons for the increase in its production is that it is not selective in terms of climate and soil. In addition, its rich nutritional value, positive effects on human health, and the long storage period and releasing time to the market when the other fruits are not abundant can be counted among the other reasons for the increases in production and consumption of pomegranate. 

The edible part of the pomegranate fruit is called aril and constitutes 45–52% of the total fruit weight. Aril consists of a white, yellow, pink, and red fleshy, juicy part and the seeds inside consist of the embryo with a shell surrounding it [5,6,7]. It has a sour or sweet taste and is also rich in bioactive compounds such as flavonoids, phenolic substances, and vitamin C. Depending on the degree of lignification, the shell surrounding the embryo is divided into four groups as hard, semi-hard, semi-soft, and soft-seeded [8]. Based on these classifications, many researchers grouped pomegranate cultivars and/or types from different regions of the world [9,10,11,12].

In recent years, the demand for pomegranate varieties with soft seeds has been increasing. They have been preferred by consumers for their ease of eating and chewing, and by the food industry for their ease of processing [13]. On the other hand, commercial pomegranate varieties are usually semi-hard or hard-seeded [10,12]. Therefore, obtaining soft-seeded pomegranate varieties with high quality and yield is one the most important goals of pomegranate breeding programs. Many studies have been carried out on the seed softness of pomegranate fruit [5,6]. Most of these studies were limited to phenotypical groupings of pomegranate cultivars and types from different regions based on the hardness of their seeds [9,10,11]. In addition, studies were also conducted to determine the possible association or relationship between seed firmness and other fruit characteristics or to develop a morphological marker related to the soft seed trait. However, no relationships were detected between soft seed trait and/or other traits studied and no molecular marker was identified for soft seed trait [5]. As a result of all these studies, it has been determined that the number of pomegranate cultivars with soft seed feature is very low. 

Many studies were carried out to determine how pomegranate seeds gain the soft-seeded trait up to date [14,15,16,17]. These studies stated that the levels of lignin and cellulose substances that make up the structure of the cell wall are an important factor on seed hardness. Some researchers [7,18,19,20] also proposed that lignification of the seed plays a key role in the determination of the hardness of pomegranate seeds. They determined that expression levels of genes responsible for lignin biosynthesis and metabolism were higher in pomegranate varieties with hard-seeded than soft-seeded. Similarly, [12] determined that lignin biosynthesis-related proteins were significantly higher in the pomegranate with hard-seeded than soft-seeded variety. On the other hand, expression level of proteins related to cellulose biosynthesis was detected higher in soft-seeded variety than hard-seeded variety at 60 days after flowering (DAF), but it was lower at 120 DAF. In addition to these results, the number of proteins involved in cell wall degradation was higher in the soft-seeded pomegranate variety than the hard-seeded variety. In another study on protein analysis, it was shown that out of 892 proteins analyzed, the expression of 76 proteins showed differences in between soft-seeded and hard-seeded pomegranate varieties [21]. Some other studies were also attempted to identifiy the differences between soft- and hard-seeded pomegranate cultivars at genome level have been conducted [22,23]. Lue et al. [22] identified two miRNAs, mdn-miR164e, and mdm-mir172b important for the regulation of the expression of genes involved in modification of the cell wall structure such as NAC1, WRKY, and MYC transcription factors. In addition, miRNA-SSR primer pairs, revealing the differences at the genome level between hard- and soft-seeded pomegranate varieties were identified. Furthermore, as a result of the comparison of the genomes of two cultivars that differ in seed firmness, some variations were observed at the genome level, as well as differences between the expressions of genes belonging to the MAPK and ABC transporter families [23]. All these results showed that the differences on seed hardness of pomegranate varieties may be due to the differences in the substances, genes, and/or proteins that are involved in the synthesis and degradation of the cell wall of the seeds. In addition to all these studies to reveal the cause of seed firmness, studies have also been conducted to determine the possible relationship or association between seed firmness and other fruit characteristics or to develop markers for soft seed trait. However, no relationships were detected between soft-seed trait and/or other traits studied and no molecular marker was identified for soft-seed trait [5,13,24]. 

Molecular markers display genetic differences between individuals with opposite traits in the form of nucleotide sequences and have been developed and used to accelerate the breeding process in many crops. This procedure is called marker assisted selection (MAS). Determination of the characteristics of the fruits could be possible without waiting for an average of 5–10 years for bearing fruits in perennial plants using MAS. Due to this time-saving process, it is also possible to reduce the space, cost, and labor [25,26]. In order to use all these advantages of MAS in pomegranate breeding as well, it is necessary to develop molecular markers associated with economically important traits in pomegranate. Therefore, in this study, molecular markers associated with the soft- and hard-seed traits in pomegranates were developed. Different molecular markers have been developed to date using different techniques, and each has different advantages. Among them, random amplified polymorphic DNA (RAPD) marker has been used for many years in many agricultural products because it can be used without the need for sequence information and requires very small amount of DNA. In this study, the RAPD technique combined with bulked segregant analysis (BSA) was used to find the marker(s) associated with soft- and hard-seed characteristics in pomegranates. 

## 2. Materials and Methods

### 2.1. Plant Material

Pomegranate genotypes were obtained from hybridization of different combinations of hard-seeded Ernar, medium-hard-seeded Hicaznar, and soft-seeded Fellahyemez cultivars in 1990 in Turkey. These crosses were made in order to develop new pomegranate varieties and phenological, morphological, and pomological characteristics of the population were determined by different researchers at different time periods [27,28] at the Western Mediterranean Agricultural Research Institute in Turkey. Among this population, 94 pomegranate genotypes and their parents were used as plant material in this study. Genotypes previously scored as hard-seeded or soft-seeded were re-scored by different experts in two harvest seasons in this study according to Yazici and Sahin [28]. Based on these evaluations, new phenotypical groups were formed and only hard-seeded and soft-seeded genotypes were used as plant material in this study.

### 2.2. DNA Isolation and Bulking

Genomic DNAs were isolated from collected leaves of soft- and hard-seeded genotypes using CTAB DNA isolation protocol developed by Doyle and Doyle [29]. The amount of isolated DNA was analyzed by NonoDrop spectrophotometer (Thermo Fisher, USA) and the quality was analyzed by 1.5% agarose gel electrophoresis. Equal amounts of genomic DNAs from 5–7 individuals showing nearly 100% soft-seeded (16–91, 16–99, 19–61, 19–66, 18–20) or hard-seeded characteristics (16–74, 17–21, 17–06, 16–03, 16–145, 16–174, 16–169) were mixed in two separate tubes. In this study, these two tubes were used for bulk segregant analysis (BSA), in which the DNAs of individuals belonging to two opposite groups in terms of seed firmness were mixed in equal amounts and bulked. 

### 2.3. Polymorphisms Analysis by RAPD and SSR Primers 

The bulked DNA samples containing genomic DNAs of pomegranate genotypes with hard- and soft-seeded were analyzed by polymerase chain reaction (PCR) method using 260 random primers (Operon Technologies Inc., Alameda, CA, USA) and 24 different SSR primer pairs previously developed for pomegranate [13,30,31,32,33,34,35,36,37,38,39]. RAPD primers were primarily selected based on previous studies in pomegranate and random primers known to give polymorphism in pomegranate fruit were used first, and then random selections were made among other Operon random primers. A list of all primers used in this study is given in Supplementary Files Tables S1 and S2. In each random primer or SSR primer pair, a PCR mix was prepared with 1X PCR buffer solution (50 mM KCl at 25 °C, 10 mM Tris HCl pH 9.0, 1% Triton X-100) 2.5 mM MgCl_2_, 0.2 mM dNTP, 10 µM RAPD primer or SSR primers, 1.25 U Taq polymerase (Thermo Fisher, USA), and 25–75 ng DNA. The PCR amplification was performed using MJ Mini PTC1148 and ICycler (Bio-Rad, USA) thermocyclers. PCR reactions were performed in thin-walled PCR plates or tubes in two different thermocyclers programmed as an initial denaturation of 5 min at 94 °C, followed by 35–40 cycles of denaturation for 30 sec-1 min at 94 °C, primer annealing for 30 sec-1 min at 35–65 °C, and primer extension for 2–3 min at 72 °C followed by one cycle of at 72 °C for 10 min for final primer extension. After the amplification, RAPD-PCR products were separated on a 1.5–3% agarose gel in 1 × TAE buffer [50 × (2 M Tris-base, 0.1 M EDTA pH 8.0, 5.7% glacial acetic acid)] and photographed under UV light using Mini BIS-Pro (DNR, Neve Yamin, Israel) gel imaging system. On the other hand, PCR products of SSR were separated using Qsep 100 fragment analyzer system (BiOptic, New Taipei City, Taiwan).

### 2.4. Scoring and Marker Analysis

Only the intense and clear bands consistently produced using genomic DNAs isolated at different time and different PCR machines were considered to be polymorphic and selected for further analysis. To confirm the consistency of a selected polymorphic bands, repetitive PCR reactions were performed using genomic DNAs of individuals constituting the bulked groups. Validation of polymorphic bands was performed using DNAs from the other members of progeny population, but not used in bulk analysis.

### 2.5. Conversion of RAPD Markers to Insertion-Deletions (InDels) Markers

Polymorphic DNA fragments associated with hard and soft seed traits were cut from agarose gel and purified using QIAquick Gel Extraction Kit (Qiagen, Hilden, Germany). The purified DNA fragments were cloned into the pGEM-T easy vector using the T-A cloning method with pGEM-T easy PCR cloning system (Promega, Madison, WI, USA), as suggested by the manufacturer’s instructions to determine their sequences. 

The clones were transferred to *Escherichia coli* JM109 cells by heat shock transformation and screening of the resulting bacterial colonies was performed by colony PCR using M13 forward (F) and reverse (R) primers located outside of the T-A cloning site. Colonies showing positive result by colony PCR were grown in liquid LB medium with ampicillin, and purification of the plasmid DNA was performed using the Mini Prep Plasmid DNA isolation Kit (Qiagen, Germany), according to the manufacturer’s instructions. For the confirmation of the presence of additional DNA in the plasmid, the purified plasmids were digested using the *EcoRI* enzyme (New England Biolab, Ipswich, MA, USA). Sequencing of plasmids containing polymorphic DNA fragments was carried out by bidirectional Sanger sequencing using universal primers, M13 F, and M13 R. Sequences of the obtained polymorphic DNA fragments were analyzed using the Vector NTI suite program (InforMax, Frederick, MD, USA). BLASTN and BLASTX applications of the National Center for Biotechnology Information (NCBI) were used to compare the sequences with the other sequences in GenBank at the nucleotide and protein level, respectively. Comparisons of the sequences with each other were performed using the BLAST-alignment module of the NCBI program. Based on the sequence comparisons, the InDel primers (forward 5′ GGCCCTCACATATTAAGTTCAC 3’ and reverse 5′ GTATCTTGAAAGTCAATGAGCC 3’) were designed. These InDel primers were used with 10 µM concentration in a 25-μL PCR reaction mixture as described in Section 2.3. The amplification of these PCR was performed in MJ Mini PTC1148 thermocycler (Bio-Rad, Hercules, CA, USA) with conditions at 94 °C for 5 min for initial denaturation for one cycle, followed by 39 cycles of 94 °C for 30 s denaturation, 55 °C for 30 s primer annealing, 72 °C for 30 s primer extension, and a final primer extension at 72 °C for 10 min. PCR products were separated and visualized as described in Section 2.3 by preparing a 3% agarose gel.

## 3. Results

**Figure 1:**
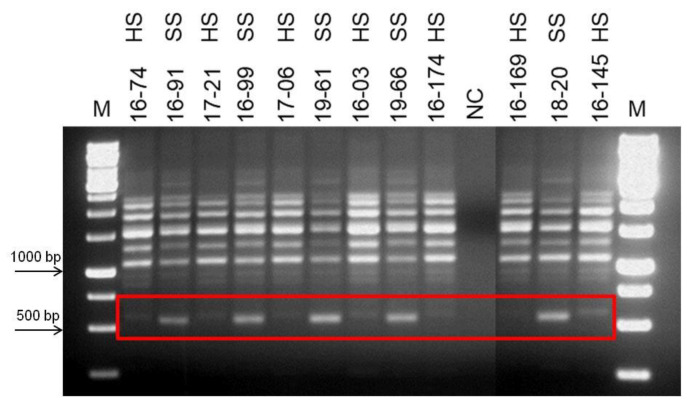
Development of RAPDHS1 and RAPDSS1 markers for distinguishing soft- and hard-seeded of pomegranate genotypes. Amplification products of OPK-12 primer. M indicates 1 kb molecular weight marker, HS indicates hard-seeded individuals, SS indicates soft-seeded individuals, and red box indicates polymorphic bands, 550 bp and 600 bp.

**Figure 2:**
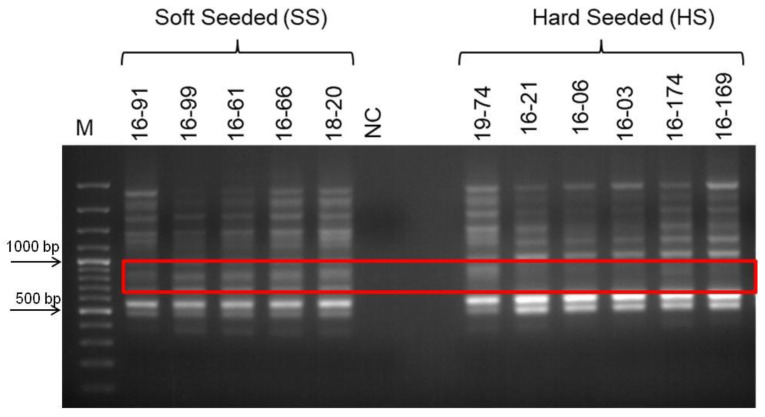
Development of RAPDSS2 marker for distinguishing the soft- and hard-seeded pomegranate genotypes. Amplification products of OPAI-08 primer. M indicates 100 bp molecular weight marker and red box indicates polymorphic band of 700 bp.

**Figure 3:**
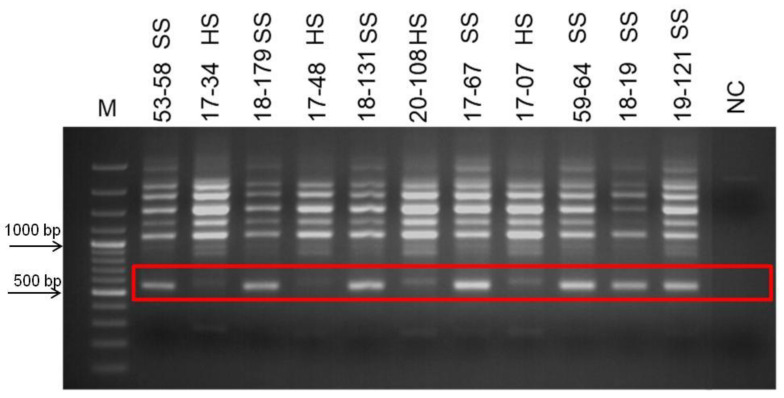
Validation of RAPDSS1 and RAPDHS1 markers using DNAs from the other soft and hard-seeded pomegranate genotypes in the population. Amplification products of OPK-12 primer. M indicates 100 bp molecular weight marker, SS indicates soft-seeded individuals, HS indicates hard-seeded individuals, and red box indicates polymorphic bands, 550 and 600 bp.

**Figure 4:**
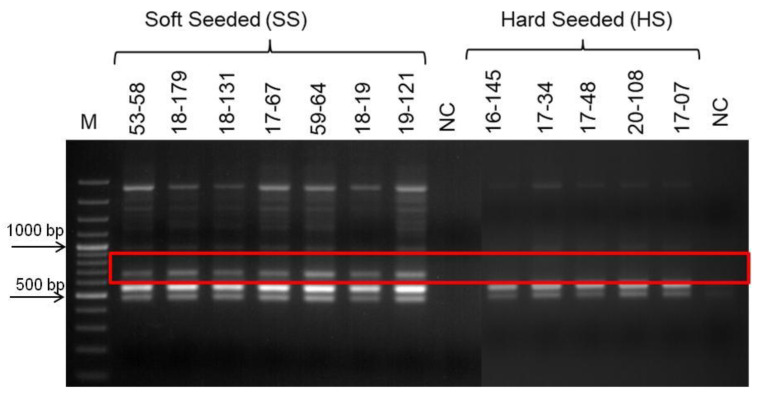
Validation of RAPDSS2 marker using DNAs from the other soft- and hard-seeded pomegranate genotypes in the population. Amplification products of OPAI-08 primer. M indicates 100 bp molecular weight marker and red box indicates polymorphic band of 700 bp.

Phenotypical groupings of the selected population based on their seed hardness were re-evaluated for two years in row in this study, and new groups were formed. Bulked DNA samples representing opposite group of individuals’, hard-seeded, and soft-seeded were formed based on this new grouping. As a result of PCR analyzes using bulked DNA samples and SSR primer pairs, no polymorphic band or marker distinguishing hard- and soft-seeded individuals was determined. However, RAPD-PCR analysis using different random primers (OPE-19, OPD-17, OPAD-18, OPAY-09, OPAE-14, OPC-12, OPR-15, OPK-12, OPAI-08) produced 10 polymorphic bands indicating the differences between two bulked samples. To test the consistency of the obtained polymorphic bands as well as their ability to distinguish between hard- and soft-seeded individuals, genomic DNAs of individuals analyzed several times by RAPD-PCR using these primers. After repeated RAPD-PCR analyses, polymorphic bands were consistently obtained only from OPK-12 and OPAI-08 primers. Moreover, the validity of these polymorphic bands was then confirmed by PCR analyses of individual genomic DNAs constituting the bulks. After multiple PCR analyses, three polymorphic bands produced by these two different primers that were able to distinguish individuals with hard- and soft-seeded pomegranate genotypes were identified. Two of these polymorphic bands, about 550 and 600 bp, were obtained from RAPD-PCR amplification using the OPK-12 primer. These two polymorphic bands were very close to each other in size, but one band was only in soft-seeded individuals with a size of 550 bp, and the other band was only present in hard-seeded individuals with a size of 600 bp band. These markers are named as RAPDSS1 and RAPDHS1 markers, respectively (Figure 1). On the other hand, only one polymorphic band was obtained as a result of RAPD-PCR analysis with OPAI-08 primer. The analyses clearly showed that the 700 bp polymorphic band was only present in individuals in the soft-seeded genotype bulks and not in the individuals forming the hard-seeded genotype bulks (Figure 2). After demonstration of its consistent ability to distinguish soft- and hard-seeded individuals from each other, this polymorphic band was named as RAPDSS2 marker. Validation of all these three molecular markers was performed by PCR reactions using DNAs from the other hard- and soft-seeded individuals which were not included in bulks in the population (Figure 3 and Figure 4). 

**Figure 5:**
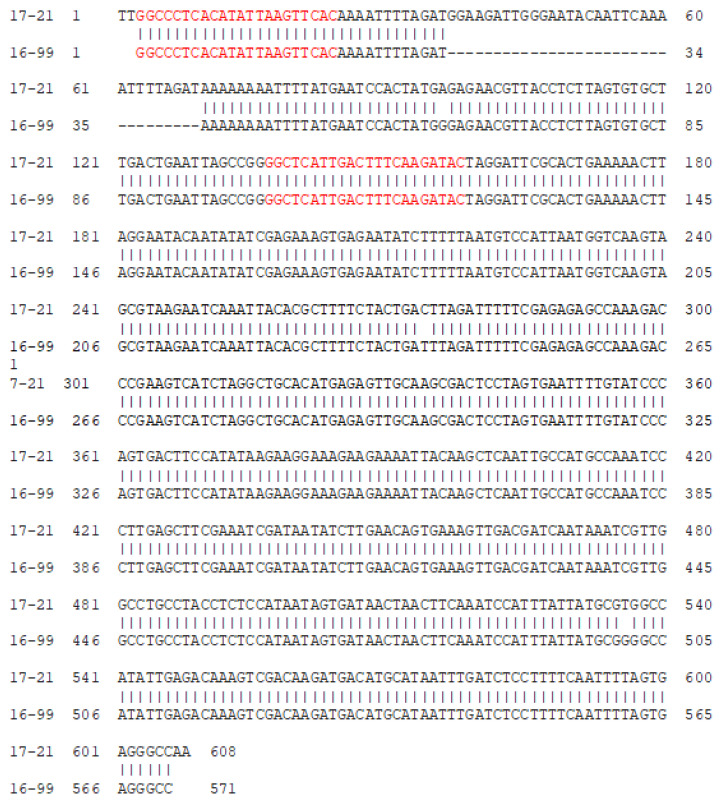
The comparison of the nucleotide sequences of hard-seeded (17–21) and soft-seeded (16–99) genotypes. The red section defines the primer sites.

The OPK-12 primer produced polymorphic bands between hard- and soft-seeded individuals. PCR product of 550 bp amplified from a soft-seeded individual (16–99) and 600 bp amplified from a hard-seeded individual (17–21) by OPK-12 primer were excised from the gel and cloned to determine their DNA sequences. After cleaning the vector sequences, 571 and 608 bp clean sequence were obtained from the cloned PCR fragments of genotype 16–99 and 17–21, respectively. The comparison of the nucleotide sequences revealed that both fragments were aligned with each other and there was 33 bp deletion in soft-seeded genotype 16–99 (Figure 5).

BLASTN analysis showed that 550 bp DNA from the soft-seeded genotype 16–99 matched with uncharacterized cDNA (GenBankacession: XM_031544412.1) of *Punica granatum* which was submitted the GenBank from sequencing the soft-seeded variety, ‘Tunisia’. In addition, the results of BLASTX analysis demonstrated that this protein was found in GenBank as protein number XP_031400272 and its function has not yet been determined. On the other hand, BLAST P analyzes demonstrated that orthologs of this protein has been functioned as ‘Calcium-dependent lipid-binding domain-containing protein’ in perennial trees, *Melia azedarach* (KAJ4722804.1), and *Salix purpurea* (KAJ6719349.1). Furthermore, based on the analysis in the *Arabidopsis* Information Resource (TAIR) database, it was determined that the ortholog of this gene is *At1g04540* and its function is defined as ‘Calcium-dependent lipid binding (CaLB domain) protein’. Studies in *Arabidopsis* have also been demonstrated that this gene is expressed during mature pollen stage, germinated pollen stage, flowering stage, petal differentiation and expansion stage, and plant embryo globular stage during plant growth and development.

**Figure 6:**
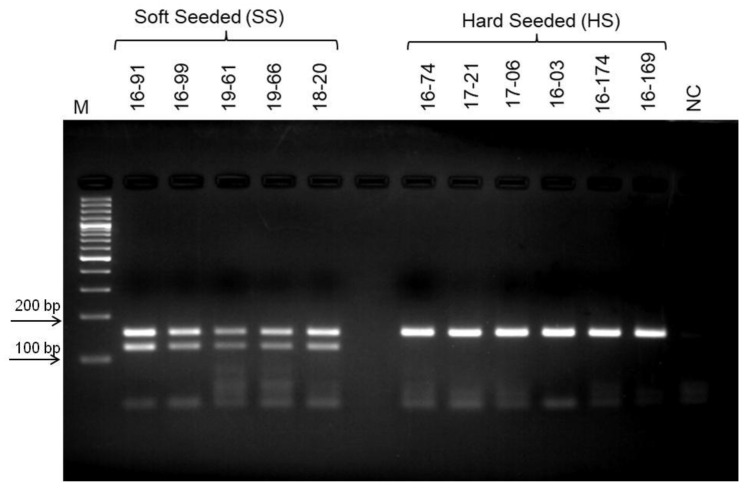
Development InDel markers for distinguishing soft- and hard-seeded of pomegranate genotypes. Amplification products InDel marker. M indicates 100 bp molecular weight marker. NC indicates negative control, SS indicates soft-seeded individuals, HS indicates hard-seeded individuals.

The sequence analysis of the soft- and hard-seeded genotypes revealed 33 bp deletion in the soft-seeded genotype. This region was the source of polymorphisms detected by OPK-12 and the sequences were identical to the coding sequences in the pomegranate genome. Based on sequence information obtained from the cloned RAPD fragments of OPK-12, two primers were designed to differentiate hard- and soft-seeded genotypes to convert the RAPD marker into more reliable sequence based markers in this study (Figure 5). Since the polymorphisms were based on internal deletion in the soft-seeded genotype, the sequence-characterized marker developed based on these sequences is considered as an insertion/deletion (InDel) marker (Figure 6).

**Figure 7:**
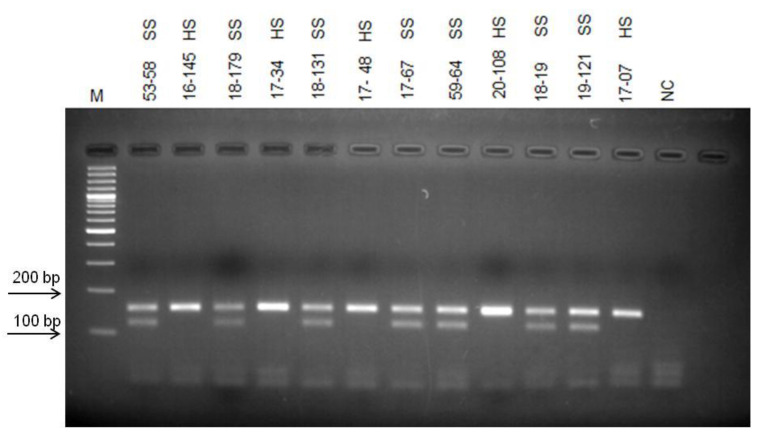
Validation of InDel marker using DNAs from the other soft- and hard-seeded pomegranate genotypes in the population. Amplification products of InDel marker. M indicates 100 bp molecular weight marker. NC indicates negative control, SS indicates soft-seeded individuals and HS indicates hard-seeded individuals.

This InDel marker was first tested by PCR analyzes of the individual genomic DNAs forming the bulks. This PCR analysis clearly showed that while only 156 bp fragment was amplified from hard-seeded genotypes, a 123 bp fragment was amplified from soft-seeded genotypes along with the 156 bp DNA fragment in the population. Later, the ability of this InDel marker to distinguish between hard- and soft-seeded individuals was tested by PCR reactions using DNAs from the other individuals’ in the population were not included in the bulk. All these results showed that the primers designed based on sequences obtained from polymorphic RAPD fragments were consistently differentiate soft- and hard-seeded genotypes in the hybrid pomegranate population (Figure 7).

## 4. Discussion

Pomegranate varieties with soft-seeded feature are very few and only found in an area limited to a few ecological regions in the world [9]. For example, it was determined that only 2 of 87 different pomegranate genotypes showed soft seed characteristics [40,41,42]. Similarly, Khadivi and Arab [11] determined that only 17 of the 70 pomegranate genotypes they examined soft-seeded trait. Similar to the results of these studies, only 12 of the 94 genotypes examined in this study showed completely soft-seed characteristics. Further, it has been observed that pomegranates with this soft-seed trait do not show the desired fruit characteristics sufficiently. This result is consistent with the results of other studies, which determined that pomegranate cultivars with soft-seeded features do not have other good fruit quality criteria [9,12]. On the other hand, pomegranates with hard-seed features generally have the desired peel and aril color. For example, Hicaznar, a pomegranate variety used in this study and also widely cultivated, has a hard-seeded cultivars although it shows superior characteristics in terms of dark red skin and other fruit characteristics. These findings show that the number of pomegranate cultivars with soft-seeded characteristics is quite low in the world and the most of them do not have other good fruit characteristics. Therefore, it is necessary to develop new pomegranate cultivars with red skin and red aryl color, and also soft-seeded features.

Development of new pomegranate varieties with desired characteristics is very difficult and takes a long period of time by conventional breeding methods. Marker assisted selection (MAS), which is among the new breeding techniques, reduce the amount of time during breeding of new fruit cultivars [43,44]. MAS allows the selection based on the genotype directly without looking at the phenotype of the plants [45]. In order to apply this technique, there is a need to develop molecular markers associated with the desired traits. 

Up to date, no molecular marker to be used directly in the breeding process of pomegranates has been identified. However, there are some studies conducted for the identification of molecular markers related with some traits including soft-seed feature. For example, in a study conducted by Sarkhosh et al., [5], a correlation was tried to be found between different fruit characteristics and RAPD markers of soft-seeded pomegranate cultivars, but no correlation was found between the fruit characteristics examined and RAPD markers. In another study, molecular markers were developed associated with important horticultural traits including seed hardness by performing quantitative trait loci (QTL) mapping [46]. Although this QTL region associated with seed firmness was found to be significant in consecutive years, it has not been validated and is thought to be specific for the population studied due to genetic differences between the parents. 

In recent years, especially after completion of the pomegranate genome, there have been a number of studies to find the differences between soft- and hard-seeded pomegranates at DNA, RNA, and protein level. In these studies, although some differences were determined at DNA, RNA, and protein levels between the compared genomes, no molecular marker that can be used in the breeding process of pomegranates has been determined yet [12,21,22,23]. In this study, three RAPD markers associated with soft- and hard-seeded traits were developed. The InDel marker was also developed based on the sequences of two of these polimorfic RAPD fragments. The ability of all these markers to distinguish soft-seeded and hard-seeded pomegranate genotypes from each other was tested. The markers developed in this study will allow soft-seeded pomegranate genotypes to be selected in an earlier developmental stage and in a shorter time during pomegranate breeding. To our knowledge, this is the first report on the identification of molecular markers that can distinguish sof-seeded and hard-seeded pomegranate genotypes.

## 5. Conclusions

In plant breeding studies, the MAS technique is used to select individuals with desired characteristics in a short time and effectively. In this study, molecular markers associated with soft- and hard-seeded characteristics were developed for the first time to use of MAS technique in pomegranate breeding studies. Among these markers, three of them were RAPD markers and the product of one of these RAPD markers were sequenced and converted to the sequence-characterized InDel marker. All the markers developed in this study will enable pomegranate breeders to select the desired soft-seeded pomegranate genotypes easily without waiting 5–6 years for bearing fruit and will ensure that the breeding process is carried out effectively and quickly. 

## Data Availability

The authors confirm that the data supporting the findings of this study are available within the article. The data are also provided in the supplementary materials, and may be shared upon request.

## Supplementary Materials

The following supporting information can be downloaded at: https://www.mdpi.com/article/10.3390/life13051123/s1, Table S1: Names and sequences of RAPD primers used; Table S2: Names and sequences of SSR primers used.
