# Supplementary material for: Development of Molecular Marker Linked to Seed Hardness in Pomegranate Using Bulked Segregant Analysis
Source: Life (Basel). 2023 May 1;13(5):1123. doi: 10.3390/life13051123 (PMC10221812; doi:10.3390/life13051123)
Supplement: Supplementary file 1 [file life-13-01123-s001.zip › Supplementary Tables S1 and S2.pdf]

Supplementary Table S1. Names and sequences of RAPD primers used

Primer Name	Sekans	Primer Name	Sekans	Primer Name	Sekans	Primer Name	Sekans
OPAY-01	GTCCACCTCT	OPS-01	CTACTGCGCT	OPR-01	TGCGGGTCCT	OPQ-01	GGGACGATGG
OPAY-02	TGCGAAGGCT	OPS-02	CCTCTGACTG	OPR-02	CACAGCTGCC	OPQ-02	TCTGTCGGTC
OPAY-03	TTTCCGGGAG	OPS-03	CAGAGGTCCC	OPR-03	ACACAGAGGG	OPQ-03	GGTCACCTCA
OPAY-04	AAGGCTCGAC	OPS-04	CACCCCCTTG	OPR-04	CCCGTAGCAC	OPQ-04	AGTGCGCTGA
OPAY-05	TCGCTGCGTT	OPS-05	TTTGGGGCCT	OPR-05	GACCTAGTGG	OPQ-05	CCGCGTCTTG
OPAY-06	GGCTTCGCAA	OPS-06	GATACCTCGG	OPR-06	GTCTACGGCA	OPQ-06	GAGCGCCTTG
OPAY-07	GACCGTCTGT	OPS-07	TCCGATGCTG	OPR-07	ACTGGCCTGA	OPQ-07	CCCCGATGGT
OPAY-08	AGGCTTCCCT	OPS-08	TTCAGGGTGG	OPR-09	TGAGCACGAG	OPQ-08	CTCCAGCGGA
OPAY-09	CCGATCCAAC	OPS-09	TCCTGGTCCC	OPR-10	CCATTCCCCA	OPQ-09	GGCTAACCGA
OPAY-10	CAAGGCCCCT	OPS-10	ACCGTTCCAG	OPR-11	GTAGCCGTCT	OPQ-10	TGTGCCCGAA
OPAY-11	ACGCGCCTTC	OPS-11	AGTCGGGTGG	OPR-12	ACAGGTGCGT	OPQ-11	TCTCCGCAAC
OPAY-12	CTGTCGGCGT	OPS-12	CTGGGTGAGT	OPR-13	GGACGACAAG	OPQ-12	AGTAGGGCAC
OPAY-13	CCGCTCGTAA	OPS-13	GTCGTTCCTG	OPR-14	CAGGATTCCC	OPQ-13	GGAGTGGACA
OPAY-14	GGTGGGTAGA	OPS-14	AAAGGGGTCC	OPR-15	GGACAACGAG	OPQ-14	GGACGCTTCA
OPAY-15	CCAAGAGGCA	OPS-15	CAGTTCACGG	OPR-16	CTCTGCGCGT	OPQ-15	GGGTAACGTG
OPAY-16	GGTGTGGTTC	OPS-16	AGGGGGTTCC	OPR-17	CCGTACGTAG	OPQ-16	AGTGCAGCCA
OPAY-17	GGTGATTCGG	OPS-17	TGGGGACCAC	OPR-18	GGCTTTGCCA	OPQ-17	GAAGCCCTTG
OPAY-18	ACCCCAACCA	OPS-18	CTGGCGAACT	OPR-19	CCTCCTCATC	OPQ-18	AGGCTGGGTG
OPAY-19	AACTTGGCCC	OPS-19	GAGTCAGCAG	OPR-20	ACGGCAAGGA	OPQ-19	CCCCCTATCA
OPAY-20	TCATTCGCCC	OPS-20	TCTGGACGGA	OPM-01	GTTGGTGGCT	OPQ-20	TCGCCCAGTC
OPK-01	CATTCGAGCC	OPL-01	GGCATGACCT	OPM-02	ACAACGCCTC	OPN-01	CTCACGTTGG
OPK-02	GTCTCCGCAA	OPL-02	TGGGCGTCAA	OPM-03	GGGGGATGAG	OPN-02	ACCAGGGGCA
OPK-03	CCAGCTTAGG	OPL-03	CCAGCAGCTT	OPM-04	GGCGGTTGTC	OPN-03	GGTACTCCCC
OPK-04	CCGCCCAAAC	OPL-04	GACTGCACAC	OPM-05	GGGAACGTGT	OPN-04	GACCGACCCA
OPK-05	TCTGTCGAGG	OPL-05	ACGCAGGCAC	OPM-06	CTGGGCAACT	OPN-05	ACTGAACGCC
OPK-06	CACCTTTCCC	OPL-06	GAGGGAAGAG	OPM-07	CCGTGACTCA	OPN-06	GAGACGCACA
OPK-07	AGCGAGCAAG	OPL-07	AGGCGGGAAC	OPM-08	TCTGTTCCCC	OPN-07	CAGCCCAGAG
OPK-08	GAACACTGGG	OPL-08	AGCAGGTGGA	OPM-09	GTCTTGCGGA	OPN-08	ACCTCAGCTC
OPK-09	CCCTACCGAC	OPL-09	TGCGAGAGTC	OPM-10	TCTGGCGCAC	OPN-09	TGCCGGCTTG
OPK-10	GTGCAACGTG	OPL-10	TGGGAGATGG	OPM-11	GTCCACTGTG	OPN-10	ACAACTGGGG
OPK-11	AATGCCCCAG	OPL-11	ACGATGAGCC	OPM-12	GGGACGTTGG	OPN-11	TCGCCGCAAA
OPK-12	TGGCCCTCAC	OPL-12	GGGCGGTACT	OPM-13	GGTGGTCAAG	OPN-12	CACAGACACC
OPK-13	GGTTGTACCC	OPL-13	ACCGCCTGCT	OPM-14	AGGGTCGTTC	OPN-13	AGCGTCACTC
OPK-14	CCCGCTACAC	OPL-14	GTGACAGGCT	OPM-15	GACCTACCAC	OPN-14	TCGTGCGGGT
OPK-15	CTCCTGCCAA	OPL-15	AAGAGAGGGG	OPM-16	GTAACCAGCC	OPN-15	CAGCGACTGT
OPK-16	GAGCGTCGAA	OPL-16	AGGTTGCAGG	OPM-17	TCAGTCCGGG	OPN-16	AAGCGACCTG
OPK-17	CCCAGCTGTG	OPL-17	AGCCTGAGCC	OPM-18	CACCATCCGT	OPN-17	CATTGGGGAG
OPK-18	CCTAGTCGAG	OPL-18	ACCACCCACC	OPM-19	CCTTCAGGCA	OPN-18	GGTGAGGTCA
OPK-19	CACAGGCGGA	OPL-19	GAGTGGTGAC	OPM-20	AGGTCTTGGG	OPN-19	GTCCGTACTG
OPK-20	GTGTCGCGAG	OPL-20	TGGTGGACCA	Y-4	GTTTCGCTCCT	OPN-20	GGTGCTCCGT
OPA-04	AATCGGGCTG	OPBD-17	GTTCGCTCCC	Y-6	GTTTCGCTCCC	OPE-01	CCCAAGGTCC
OPA-19	CAAACGTCGG	OPBC-08	GGTCTTCCCT	Y-9	CTGCTGGGACA	OPE-02	GGTGCGGGAA
OPH-07	CAAACGTCGG	OPH-18	GAATCGGCCA	Y-22	GGACCCAACCT	OPE-03	CCAGATGCAC

OPH-08	GAAACACCCC	OPY-06	AAGGCTCACC	Y-30	GTGTGCCCCAC	OPE-04	GTGACATGCC
OPAD-10	AAGAGGCCAG	OPY-07	AGAGCCGTCA	Y-34	AAGCCTCGTCT	OPE-05	TCAGGGAGGT
OPAG-08	AAGAGCCCTC	OPY-11	AGACGATGGG	Y-48	ACGACCGACAC	OPE-06	AAGACCCCTC
OPAG-12	AAGAGCCCTC	OPY-13	GGGTCTCGGT	Y-51	TGGTGGCGTTA	OPE-07	AGATGCAGCC
OPAG-20	CTCCCAGGGT	OPBA-03	GTGCGAGAAC	Y-54	TGGTGGCGTTC	OPE-08	TCACCACGGT
OPAH-16	TGCGCTCCTC	OPBA-06	GGACGACCGT	Y-57	ACCCCCGACTA	OPE-09	CTTCACCCGA
OPAH-19	CAAGGTGGGT	OPBB-09	AGGCCGGTCA	D-5	GTCAGAGTCCT	OPE-10	CACCAGGTGA
OPAH-02	GGCAGTTCTC	OPBB-13	CTTCGGTGTG	OPAD18	ACGAGAGGCA	OPE-11	GAGTCTCAGG
OPAI-08	GGAAGGTGAG	OPAC-11	CCTGGGTCAG	OPAE14	GAGAGGCTCC	OPE-12	TTATCGCCCC
OPAI-18	AAGCCCCCCA	OPAD-02	CTGAACGCTG	OPAH20	CACTTCCGCT	OPE-13	CCCGATTCGG
OPB-01	TCGCAGCGAG	OPAD-04	GTAGGCCTCA	OPAJ08	TCGCGGAACC	OPE-14	TGCGGCTGAG
OPB-12	TGATGGCGTC	OPAD-13	GGTTCCTCTG	OPAJ14	GTGCTCCCTC	OPE-15	ACGCACAACC
OPB-20	CCTTGACGCA	OPAD-16	AACGGGCGTC	OPAK19	ACCGATGCTG	OPE-16	GGTGACTGTG
OPD-17	GGACCCTTAC	OPAE-10	CTGAAGCGCA	OPA02	TGCCGAGCTG	OPE-17	CTACTGCCGT
OPX-19	TTTCCCACGG	OPB-10	CTGCTGGGAC	OPA05	AGGGGTCTTG	OPE-18	GGACTGCAGA
OPBA-06	GGACGACCGT	OPAD-15	TTTGCCCCGT	OPA10	GTGATCGCAG	OPE-19	ACGGCGTATG
OPBB-03	TCACGTGGCT	OPP-02	TCGGCACGCA	OPA13	CAGCACCCAC	OPE-20	AACGGTGACC
OPBB-04	ACCAGGTCAC	OPP-14	CCAGCCGAAC	OPB05	TGCGCCCTTC	OPC08	TGGACCGGTG
OPBB-07	GAAGGCTGGG	MG-01	AGCGCCGACG	OPC05	GATGACCGCC	OPC10	TGTCTGGGTG
OPBB-08	TCGTCGAAGG	MG-11	AGGAGCTGCC	OPC06	GAACGGACTC	OPC12	TGTCATCCCC
OPBB-10	ACTTGCCTGG	MG-16	GAAGAACCGC			OPI18	TGCCCAGCCT
OPBD-07	GAGCTGGTCC					OPR08	CCCGTTGCCT

Supplementary Table S2. Names and sequences of SSR primers used.

Primer Name	Nucleotide Sequence	Primer Name	Nucleotide Sequence
ABRII-MP26-F	5-TTTCTCGAAGAATTGGGTAA-3	FM994087-F	5-GCAAAGGAAACAAAAACAAA-3
ABRII-MP26-R	5-CTGAGTAAGCTGAGGCTGAT-3	FM994087-R	5-TGATTTGATCCTCAGCTTCT-3
ABRII-MP28-F	5-ATCCTCTGTCTTTGTGTTCG-3	FM994097-F	5-ATGAATGAGGAAGACGAAAA-3
ABRII-MP28-R	5-TGAGTAATTCCGGTCAGAAG-3	FM994097-R	5-GTGCTCCATCCATACAAAAT-3
ABRII-MP42-F	5-GAGCAGAGCAATTCAATCTC-3	FM994094-F	5-GCCTATCTCGTGATCACATC-3
ABRII-MP42-R	5-AACAATTTCCCATGTTTGAC-3	FM994094-R	5-AATGGGAGCGGACTAACTAT-3
EPS01-F	5-TCTATTCCACATAGAAAGAGGGG-3	FM994095-F	5-GGACTAGCACAACTCGTAGC-3
EPS01-R	5-ATGATGTCTATGCAATTGGCTG-3	FM994095-R	5-CAACAAAATGAGAAGGTGGT-3
EPS03-F	5-CGCTGGTCACACTACTTACTCG-3	pg4-F	F: CTGATGTAATGGCTGAGCAAA
EPS03-R	5-TTGTAGTGGAAGACACAGCAGC-3	pg4-R	R: GCACTTGAACAAAGAGAATGC
EPS04-F	5-AAAGGGGAAAAAGACGAAGAAG-3	pg6-F	F: GGTTGCTCATCCCTTGACTC
EPS04-R	5-CCCTGTCCTTAAGTCTGAGTGG-3	pg6-R	R: GCGTCTGTCAGTGTCTTAGGC
EPS05-F	5-TTGTTGGGTATTCCTCTTCTC-3	pg8-F	F:CACCATAGACTTAAACGAGCACAA
EPS05-R	5-ACATCATACACCTTGCCCTC-3	pg8-R	R: GAAGCTCCATTGCCTCGTCC
EPS06-F	5-AAATCGCATCCCTCCGTCT-3	pg17-F	F: CATCAGACTACGATGGCACT
EPS06-R	5-CTGTTCGCCAGGGTAAAGA-3	pg17-R	R: GCATAATAGCCTTCAATTTACA
EPS08-F	5-TTCCCGAGAAAGTTGCATATCT-3	pg18-F	F: TCTAAGGGCAGAATGGCACT

EPS08-R	5-TAGTCCGTGAGGATTTTGTCCT-3	pg18-R	R: TGGCACTAGATCCGTAAATCT C
EPS09-F	5-TAATCCCATTCCAAACAAGTCC-3	PGCT006-F	F:TTGAATTGATGTAACGCTTG
EPS09-R	5-ATATTGACGGAGGCTTCACTGT-3	PGCT006-R	R:GAGGAAAGTCGTTTGAAGTG
EPS10-F	5-TAGCACAGGGGAAATCTGAAAT-3	PGCT015-F	F:GACGCCTTTAGTTTGCTCCA
EPS10-R	5-GGAAGAGTTTGGTTCAGGATTG-3	PGCT015-R	R:CTCGGGACAGGACTTGGAAT
EPS19-F	5-TGGGGATTATCGTTGTCTTCA-3	PGCT033-F	F:TAATAAGCTGCCCCGAAGTC
EPS19-R	5-TCCAAGCTGAACTCGTTCCT-3	PGCT033-R	R:CGGTGATGTCCCTATTGGAG
